# Language and Communication Impact of Hypertension: A Qualitative Study

**DOI:** 10.1155/2021/9931873

**Published:** 2021-07-08

**Authors:** Cecilia Amponsem-Boateng, Timothy Bonney Oppong, Weidong Zhang, Tanko Abdulai, Jonathan Boakye-Yiadom, Lianke Wang, Emmanuel Kumi Duodu Kyere

**Affiliations:** ^1^Department of Epidemiology and Biostatistics, College of Public Health, Zhengzhou University, Zhengzhou, Henan 450001, China; ^2^Komfo Anokye Teaching Hospital, Kumasi, Ghana

## Abstract

*Background*. Hypertension (HTN) is the second main source of outpatient morbidity in Ghana, and the understanding of a disease is necessary for its prevention and management. Language and communication are contributing factors to HTN in Ghana. No studies have been conducted to assess knowledge/awareness of HTN (in the context of its understanding) among students in Ghana. Following a local name for HTN in Ghana, researchers interviewed students through a focus group to assess their understanding/perception (meaning, cause, and prevention) of the disease. Available literature has concerned itself with clients' knowledge of their condition (diagnosis) rather than their comprehension of the true nature of what HTN is. The objective of this study is to assess the knowledge/awareness of HTN in the context of its understanding of the meaning, perception, causes, and prevention of hypertension among students of Ghana's Senior High School (Second Cycle). Semistructured interviews with the use of the theme lists were employed. Focus group conversations and interviews were held in the local Akan (Twi) language, which was later translated, interpreted, and analyzed. Overall, 25 second-cycle students participated. 60% were between 15 and 17 years, 24% were ≥18 years, and 16% were <15 years of age. Males were 44% and females were 56%. Students gave diverse perceptions of their knowledge of HTN. The local language's translation of HTN has influenced and affected its meaning/understanding among some, thus affecting their perception of causes and prevention.

## 1. Introduction

Hypertension is a lethal condition that kills almost 70% of people with cardiovascular disease in lower- and middle-income countries (LMICs) [1]. HTN is the major source of outpatient morbidity in patients aged 45 years and above in Ghana. A current meta-analysis found a prevalence range of 2.8% to 67.5% of HTN defined as 140/90 mm/Hg and a pooled prevalence of 27% among adults [2]. A current nationally cross-sectional survey also found a prevalence of 19.91% of HTN and 26.07% of prehypertension among students who were aged 12 to 22 years in second-cycle schools in Ghana [3]. The lack of official awareness, knowledge disparities, and inaccessibility to world health information services have been described as causes that influence the comprehension of HTN in Ghana [4]. The effect of culture on well-being is immense. Culture impacts beliefs on health, sickness, and death. It also influences notions regarding the origins of disease, strategies of healthcare, how sickness and pain are felt and expressed, when people receive support, and also the essence of what recovery people desires [5]. The vocabulary of a group of people is embedded in their culture and can characterize their perception of an illness. Conveying the concept of an illness to people can contribute to their static views on it rather than identifying it as a physical functioning feature [6].

The achievement of decent clinical practice and prevention of diseases cannot be practicable without a clear knowledge of disease communication on the part of public health promoters/practitioners, doctors, and staff clinicians [7]. Language evolves as we think about critical health problems because language can imitate by causing anxiety and dishonor and remove the attention of people from precautionary care [8]. HTN in the younger generation is not as common as in the elderly; however, youthful HTN is a major field that needs attention as some studies have demonstrated an increased prevalence of HTN in obese adolescents and infants [9, 10]. Health promotion helps individuals to improve their health and the effectiveness of health promotion through environmental empowerment and multisectoral intervention increases good activities [11]. The stage of young adulthood is typically the robust period of life and the estimated time for the onset of critical health and social well-being problems [12]. As susceptibility to HTN is not limited to old age, and since studies have proven that cardiovascular (CVD) risks in the youth progresses into adulthood and increases adulthood CVDs [13, 14], knowledge/awareness on HTN with students at this stage becomes particularly important. This is because the youth can be good agents of health promotion to their peers, family, and friends. Additionally, they are growing to represent the next adulthood population and their proper understanding of a disease is helpful for future adulthood prevalence. Existing literature on knowledge/awareness of HTN in Ghana so far was based on patient's knowledge of their condition (diagnosis) [15] rather than their understanding of the true nature of what HTN is. This study, therefore, assesses the knowledge/awareness of HTN towards an understanding of its meaning, perception, causes, and prevention among students in second-cycle schools in Ghana.

## 2. Materials and Methods

### 2.1. Design

Focus group interviews were conducted in three different regions of Ghana. Respondents were recruited from three different schools in each region at particular scheduled times. The inclusion criterion was second-cycle students in either grade 1, 2, or 3. Questions for the interviews were developed by researchers using simple unambiguous terminologies and were subsequently independently reviewed by E. A. G to check for reliability and consistency.

### 2.2. Ethical Consideration

Written informed consent was sought from the parents of students under 18 years and verbal informed consent from those 18 and above. Written information was also sent to heads of schools whose students were participating in the study, while ethical approval was attained from Zhengzhou University and the School Health and Education Programs Directorate of the Ghana Education Services.

### 2.3. Conducting the Qualitative Interview

Respondents were welcomed and made to feel relaxed. They were introduced to the interview and its goal was communicated to them. Respondents were free to move around in the hall and also help themselves with some refreshment. One person was allowed to speak at a time for clarity, and there were no right or wrong answers. They were informed about the audio taping of the sessions and the handwritten notes that would assist us to double check for accuracy. Respondents were then encouraged to avoid side discussions and turn phones off, if any. Discussions and interviews were conducted in serene locations. The lead interviewer, CAB, and the assistants, TBO and JBY, were all natives who could read, write, and understand the local language (Akan). This language was used because it is widely spoken in the chosen regions in Ghana. The interview was driven with open-ended questions and was audio-recorded. The guide was initially written in English and translated to the local Akan language for the convenience of the participants, and the respondents were free to express themselves in either Akan or English.

The guide for the interview addressed a variety of topics, such as the meaning/understanding or perception of HTN, perceived causes/risks of HTN, and how it can be prevented. All participants were contacted in their respective schools as a reminder of the upcoming study a day before the meeting for confirmation. All session interviews were done by CAB, TBO, and JBY; they were audiotaped and transcribed verbatim. The interviewers assured respondents of the confidentiality and anonymity of their identities. All interviews were conducted in halls and sessions lasted between 40 and 60 minutes in each of the regions.

### 2.4. Data Analysis

Analysis of the data was carried out using the six steps of the Braun and Clakes [16] thematic study:

1. Three researchers (CAB, TBO, and JBY) familiarized themselves with the data by transliterating the recorded interviews verbatim and constantly listening to and reading the data collected from the three groups.
2. CAB, TBO, and JBY coded the first nine interviews (G1) separately by carefully analyzing the details and generating the original codes. Three independent interviews were compared and then combined. Two researchers coded the remaining interviews and were reviewed by another researcher. The coding was matched and debated before consensus was reached. The same procedure is replicated in the three interviews.
3. Different codes were divided into possible themes and debated by four writers (CAB, TBO, JBY, and AT) before consensus was reached on potential themes.
4. The decided future topics have been checked and thematic maps have been drawn up. The data have been re-read to ensure that the thematic chart represents the data gathered. The writers addressed the issue as to whether data saturation had been achieved.
5. The resulting ideas were distilled and a description of the data was explored and considered by CAB, TBO, JBY, LW, AT, EKDK, and WZ.
6. Final analysis was carried out and illustrative examples were chosen and translated into English in order to address the study question and compare our analysis with current literature. The goal of this study was to put together the concept of HTN on the meaning/understanding, perception on causes, and prevention in its local name among Ghanaian students and to update management of HTN education among young people. Participants were not involved in the formulation of the research issue, the procurement of the design, or the conduct of the test.

## 3. Results

A total of twenty-five students (56% males, 44% females) from the three regions comprising (G1: 9; G2: 8; G3: 8) were included in this analysis. Among them, 56% were aged 15–17 years, 28% ≥ 18 years, and 16% < 15 years.

Table 1 depicts the summary of their characteristics below.

### 3.1. Overview

**Figure 1:**
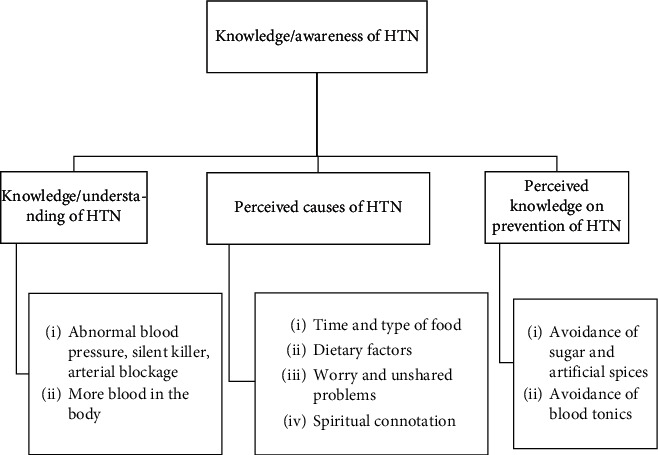
Underlying themes and subthemes.

In the interview conducted, students expressed their understanding of hypertension in the local Akan language (Mmogya mmoroso) to wit “more blood in the body.” Overall, within the three regions, which were categorized into Group1, Group 2, and Group 3 (G1: Ashanti; G2: Brong Ahafo; G3: Eastern Region), three underlying themes, and eight subthemes emerged from the interview, as shown in Figure 1. The underlying themes are knowledge/awareness or understanding of HTN definition; perceived causes and risks; perceived knowledge on prevention. Figure 1 illustrates the underlying themes and subthemes and Supplementary File1 also presents the coding of the interview results.

### 3.2. Knowledge/Awareness (Understanding of the Meaning of HTN Definition)

Two subthemes emerged from the meaning/understanding of HTN. Students' expressions on the disease were diverse. While some had some general knowledge about HTN, others had no idea and some gave wrong definitions to its meaning.

#### 3.2.1. Abnormal Blood Pressure Readings, Silent Killer, and Arterial Blockage

Some students' definitions of HTN showed they understood what the disease was. For example, on the question about explaining what HTN is, some respondents explained:

“We know HTN is also known as abnormal blood pressure when the systolic reading (which is the top reading) of a person is more than 130 and the diastolic reading (which is the down reading) is more than 90:” (P1).

“Hypertension is a disease that kills people slowly, before their family gets to know” (P4).

“HTN simply is high blood pressure readings of an individual and it can be caused by the blockage of the blood vessels because they become choked and puts more pressure on the heart to pump blood to other parts of the body” (P12).

“Well, I know it to be a heart disease and a slow killer” (P14).

“It is abnormal blood pressure for adults” (P21).

#### 3.2.2. More/Excess Blood in the Body (“Mogya Mmoroso” in the Local Language)

Response on the definition/explanation of some respondents, however, indicated that the locally coined name of HTN (“Mogya Mmoroso“) in the Akan language and by which HTN is locally called in Ghana has negatively impacted the meaning of what the disease is. For instance, the following are some of the responses:

It is “more blood in the human body” (P5; P10, P11; P17). Another added, “I do not even understand why our hospitals should get short of blood when we have a lot of people with HTN in the society” (P5).

“Well, for me, I know it happens when the body has blood in excess. So, the type of food that gives us more blood should be reduced as we age so that we will not get hypertension“ (P18).

### 3.3. Perceived Causes/Risk

On the perception of the causes of HTN within respondents, four (4) subthemes emerged from the interview.

#### 3.3.1. Time and Type of Food

Some respondents believed that the type of foods eaten at a particular time can expose someone to the risk of HTN. Some students expressed the view that eating heavy foods late at night can cause HTN since digestion is not as effective at night as during day time. Some responded as follows:

“Most people who have HTN are late eaters” (P4; P6; P9; P25; P20), and “they do not also like to drink maybe tea or eat maybe some small fruits to sleep because they are too hungry” (P25, P4). “They like to eat their heavy foods to get full and go to bed right after that, so even when they are asleep, their bodies are still working like day time” (P6; P20).

“For me, I know hypertension is pressure, so if there is too much pressure like work and exams periods you can become stressed and get hypertension“ (P22).

“We eat heavy foods during supper time. So, the heart overworks at night and may cause HTN” (P12; P14).

#### 3.3.2. Dietary Factors

Some respondents attributed the eating of synthetic and canned foods to HTN. For example, some respondents had this to say:

“There are too many synthetic oils in our meals nowadays and I think they can cause HTN” (P3; P15).

Others also think some foods and blood tonics can risk/cause HTN, as they responded with the following:

“Per its definition, I think we should reduce foods that produces more blood” (P18; P5).

“I believe HTN can be caused by taking blood tonics” (P17, P11).

#### 3.3.3. Worry and Unshared Problems

“When we have problems and we do not tell others we can get hypertension. Especially, our mothers. Most of them think too much“ (P16).

#### 3.3.4. Spiritual Connotation to Hypertension

Additionally, as evidenced in some of the following responses, there were respondents who asserted that hypertension has a spiritual connotation:

“‘As for HTN, it's a spiritual disease. Like diabetes, if you get it, you have to fast and pray for God to intervene, otherwise, it will be with you forever and if you get this HTN, you are likely to get diabetes too in the future“ (P8).

“This disease I believe can be bought spiritually and given to someone for it to manifest physically, so I think when they tell you at the hospital that you have it, the first place to go is the Church house“ (P7).

### 3.4. Perceived Knowledge on Prevention

Respondents had different ideas on how HTN can be prevented. Though some believed that eating a bit early before bedtime, reducing the intake of oily and fatty foods, and exercising are better ways of preventing HTN, others had different perceptions on how it can be prevented.

#### 3.4.1. Avoidance of Sugar and Artificial Spices

Some were of the view that avoiding sugar and artificial spices can help in the prevention of HTN. For example, some students responded with the following:

“When we stop eating sugar HTN will reduce because of the relationship it has with diabetes“ (P24).

“For me, I believe artificial spices are the causes of hypertension in our time now. Most foods are artificially spiced, even at the school's kitchen, but we cannot complain? If we stop or reduce artificial spices, it can reduce HTN and even diabetes“ (P23).

#### 3.4.2. Avoidance of Blood Tonics

“I do not actually know the meaning of the disease, but per its local name it suggests too much blood is a problem, so I think blood tonic should be avoided because it can give you more blood and then HTN will follow” (P20).

“As for me, I suggest we eat foods to get blood rather than blood tonics, because some of them even when you take, your heart beats faster” (P19).

## 4. Discussion

This study assessed the knowledge/awareness of HTN in the context of its comprehension, perception, causes, and prevention from the perspective of students in three different focus group interviews within three regions of Ghana. Three regular themes with eight subthemes were identified from the three groups, with each group representing one region: knowledge/awareness or understanding of HTN definition; perceived causes and risks; perceived knowledge on prevention. Paradoxically, the locally coined language of HTN, which is “Mogya Mmoroso” and literally translates as “more blood” in Ghana, has affected the minds of many young adults and may not be too different within the adult population since that is the known name of the disease across the country. Though some students were able to define HTN as stated by the JNC 7 [17], this study has highlighted a very important area that needs the attention of health promoters, health workers, and clinicians. It indicated the fact that in addressing serious health issues, language makes a difference [8], and caution is necessary for coining a local name for a disease. It emphasizes that simple modification in terminology and the conceptualization of disease have consequences for many areas, including medical communication with the public, advertising, and public policy [18]. Again, this demonstrates that misconception of disease is a serious matter, likely to cause harm in society. It is therefore important that more attention is given to culturally competent health services, promoters, and health professionals who understand the concept of a disease in a population before it is communicated to the general public. Ultimately, respondents in this population are not responsible for their different understandings of HTN as that was the conveyed meaning that was portrayed by the health promoters who communicated the meaning to the community. Language embodies implicit reasoning and gives meaning to what one may be trying to deliver and talk about [19].

On the perceived causes of HTN, students believed that time and type of foods eaten can cause HTN. Though scholarly work in connection with this is limited, a recent study at the meeting of the American Heart Association confirmed that eating high-calorie meals after 6 PM significantly increases the risk for high blood pressure [20]. Largely, most Ghanaians are used to eating heavy meals late in the evening (after 6 PM), and the high prevalence rate of HTN in Ghana may have a link to the time and the type of foods eaten. Additionally, limited or no study so far has highlighted synthetic and canned foods as causes to HTN. The Dietary Approach to Stop HTN (DASH) diet, however, recommends the avoidance of canned, fast, and processed foods. This is an indication of a possible link between synthetic and canned foods to HTN and education is needed in the reduction of its consumption.

Once more, worry and unshared problems, as elaborated by students, can cause HTN. Studies have linked exposure to chronic and psychosocial stress to HTN [21, 22]. This linkage among mothers (females), however, may have other influential factors such as the traditional role of women in Ghana where they are kept in households to care for children and keep the house in order and spousal abuse, which is more common in women who choose to follow a career outside the home. Although no study has affirmed any spiritual aspects of HTN, this study has emphasized this assumption in the Ghanaian community and probably among most Africans. Ghanaians are religious at the core and attach spirituality to daily happenings. Alongside the African traditional religious beliefs, culture, and practices, there is no doubt in the attachment of spirituality to HTN by some students. On the perceived knowledge on prevention of HTN, however, the mention by some students of the avoidance of sugar, artificial spices, and blood tonics may necessitate the attention of stakeholders.

This study is particularly important since (1) it is the first study to assess knowledge/awareness of HTN based on the understanding of the disease; (2) the assessment (interview) was done in three different geographical locations in the country; (3) it has highlighted factors that were never considered with HTN that needs attention; (4) it has elaborated on a topic in a group that has not been targeted as far as HTN is concerned. To curb this misconception among the targeted group, it is recommended that the Ghana Health Services, in collaboration with the Ministry of Education, makes an amendment in the curriculum of schools, starting from the basic to senior high schools where students can be properly communicated to on diseases. Public Health education on televisions, radios, and local community information centres on HTN and other diseases is also recommended to curb these misconceptions from the general public.

In conclusion, this study has focused on a topic that has not as yet been touched on in a younger population in Ghana. It has revealed that early and proper understanding of a disease is crucial for effective prevention as measures are put in place for either individuals or populations. This study is beneficial as it enlightens public health workers, health promoters, and stakeholders to take precautionary measures in the future regarding the coining of names for diseases in local/native languages. Intensive Community Health Education programs are needed to curb the misconception of HTN at the district levels in Ghana. Additionally, students should be taught in schools to increase their health literacy as the proper knowledge and understanding of disease among the youth will empower them to become good publicity agents in health promotion.

## 5. Summary Table

### 5.1. What Is Known about the Topic

- Culture impacts beliefs on health, sickness, and death.
- Health promotion helps individuals to improve their health.

### 5.2. What This Study Adds

- Locally coined language of a disease in health promotion impacts its understanding to the general public.
- The proper understanding of a disease is crucial for effective prevention.

## Figures and Tables

**Table 1:** Summary of participant characteristics.

Participants (*p*)	Sex	Type of school	Age	Education level	Level of study	Major course of study	Profession of parent
P1	Female	Public/govt	17	SHS	2	Home economics	Teacher
P2	Male	Public/govt	17	SHS	3	Science	Trader
P3	Male	Private	16	SHS	2	Agriculture	Teacher
P4	Female	Private	16	SHS	2	Home economics	Nurse
P5	Male	Public/govt	19	SHS	3	Science	Driver (professional)
P6	Female	Public/govt	17	SHS	2	Agriculture	Trader
P7	Female	Public/govt	14	SHS	1	General arts	Hairdresser
P8	Female	Public/govt	15	SHS	1	Science	Trader
P9	Female	Private	16	SHS	3	Science	Artist
P10	Male	Public/govt	15	SHS	2	General arts	Carpenter
P11	Female	Private	14	SHS	1	Home economics	Trader
P12	Male	Public/govt	18	SHS	2	Business/accounting	Medical assistant
P13	Male	Public/govt	16	SHS	1	Science	Farmer
P14	Female	Public/govt	14	SHS	1	Science	Matron
P15	Female	Public/govt	15	SHS	1	Science	Photographer
P16	Male	Private	20	SHS	3	General arts	Teacher
P17	Male	Public/govt	17	SHS	2	Agriculture	Food vendor
P18	Female	Public/govt	19	SHS	3	Business/accounting	Driver (professional)
P19	Female	Public/govt	18	SHS	3	Business/accounting	Businesswoman
P20	Male	Public/govt	19	SHS	3	Business/accounting	Farmer
P21	Female	Public/govt	14	SHS	1	Home economics	Hairdresser
P22	Female	Private	16	SHS	2	General arts	Trader
P23	Male	Public/govt	17	SHS	2	General art	Teacher
P24	Male	Public/govt.	19	SHS	3	Science	Masonry
P25	Female	Private/govt.	17	SHS	2	Science	Businessman

## Data Availability

The datasets used and/or analyzed during the current study are available from the corresponding author on reasonable request.

## Supplementary Materials

Supplementary Materials S_1: Coding of interview results.

## References

[1] Hien H. A., Tam N. M., Tam V., Derese A., Devroey D. (2018). Prevalence, awareness, treatment, and control of hypertension and its risk factors in (central) vietnam. *International Journal of Hypertension*.

[2] Bosu W. K., Bosu D. K. (2021). Prevalence, awareness and control of hypertension in Ghana: a systematic review and meta-analysis. *PLoS ONE*.

[3] Amponsem-Boateng C., Oppong T. B., Zhang W. (2021). Screening of hypertension, risks, knowledge/awareness in second-cycle schools in Ghana. A national cross-sectional study among students aged 12-22. *Journal of Human Hypertension*.

[4] Agyei-Baffour P., Tetteh G., Quansah D. Y., Boateng D. (2018). Prevalence and knowledge of hypertension among people living in rural communities in Ghana: a mixed method study. *African Health Sciences*.

[5] “Culture Clue ™ for Clinicians UWMC Patient and Family Education Services Communication Guide: All Cultures Establishing Trust Respecting Differences Providing Culturally Acceptable Care.“ [Online]. Available: http://www.ethnomed.org

[6] Warner R. (2019). The relationship between language and disease concepts. *The International Journal of Psychiatry in Medicine*.

[7] Wilkinson S. R. (2003). *Coping and Complaining*.

[8] George D. R., Whitehouse E. R., Whitehouse P. J. (2016). Asking more of our metaphors: narrative strategies to end the “war on alzheimer’s“ and humanize cognitive aging. *The American Journal of Bioethics*.

[9] Redwine K. M., Daniels S. R. (2012). Prehypertension in adolescents: risk and progression. *The Journal of Clinical Hypertension*.

[10] Falkner B. (2012). Prehypertension in adolescents: how high is the risk for hypertension?. *The Journal of Pediatrics*.

[11] “WHO | NCDs, Poverty and Development,“ *WHO*, 2016

[12] Park M. J., Scott J. T., Adams S. H., Brindis C. D., Irwin C. E. (2014). Adolescent and young adult health in the United States in the past decade: little improvement and young adults remain worse off than adolescents. *Journal of Adolescent Health*.

[13] Jolliffe C. J., Janssen I. (2006). Vascular risks and management of obesity in children and adolescents. *Vascular Health and Risk Management*.

[14] Amponsem-Boateng C., Zhang W., Oppong Bonney T., Opolot G., Kumi Duodu Kyere E. (2019). A cross-sectional study of risk factors and hypertension among adolescent Senior High School students. *Diabetes, Metabolic Syndrome and Obesity: Targets and Therapy*.

[15] Jolles E. P., Padwal R. S., Clark A. M., Braam B. (2013). A Qualitative Study of Patient Perspectives about Hypertension. *International Scholarly Research Notices*.

[16] Braun V., Clarke V. (2006). Using thematic analysis in psychology. *Qualitative Research in Psychology*.

[17] Nhlbi P. (2003). Detection, evaluation, and treatment of high blood pressure the seventh report of the joint national committee on complete report. *JAMA*.

[18] Young M. E., Norman G. R., Humphreys K. R. (2008). The role of medical language in changing public perceptions of illness. *PLoS One*.

[19] Krisberg K. (2018). What’s in a word? How language affects public health: research shows word choices can influence well-being, treatment. *Nations Health*.

[20] “Late Night Eating and Your Heart - Cleveland HeartLab, Inc.” https://www.clevelandheartlab.com/blog/late-night-eating-and-your-heart/ (accessed Aug. 09, 2020)

[21] Cuffee Y., Ogedegbe C., Williams N. J., Ogedegbe G., Schoenthaler A. (2014). Psychosocial risk factors for hypertension: an update of the literature. *Current Hypertension Reports*.

[22] Spruill T. M. (2010). Chronic psychosocial stress and hypertension. *Current Hypertension Reports*.

