# Supplementary material for: Language and Communication Impact of Hypertension: A Qualitative Study
Source: Int J Hypertens. 2021 Jul 8;2021:9931873. doi: 10.1155/2021/9931873 (PMC8282399; doi:10.1155/2021/9931873)
Supplement: Supplementary Materials — S_1: Coding of interview results. [file 9931873.f1.docx]

| **First-order codes** | **Second-order categories** | **Aggregated**  **themes** |
| --- | --- | --- |
| *‘we know HTN is also known as abnormal blood pressure when the systolic reading (which is the top reading) of a person is more than 130 and the diastolic reading (which is the down reading) is more than 90 (P1).  *‘It is abnormal blood pressure for adults’ (P21).  * HTN is when the pressure of the blood in the body is not normal (p 25).  *HTN is Simply abnormal Blood pressure (P3) | Abnormal Blood pressure | Knowledge/  understanding of HTN |
| *‘Hypertension is a disease that kills people slowly, before their family gets to know’. (P4)  ‘*well, I know it to be a heart disease and a slow killer’ (P14)**.**  *HTN kill people slowly because sometimes people don’t know they have it (P 9). | Silent killer |  |
| *HTN simply is high blood pressure readings of an individual and it can be caused by the blockage of the blood vessels because they become chocked and puts more pressure on the heart to pump blood to other parts of the body’ (P12).  *This disease happens when the arteries of someone is blocked (P15).  * This is a disease caused by chocked arteries (P13) . | Arterial blockage |  |
| *it is ‘more blood in the human body’ so, I don’t even understand why our hospitals should get short of blood when we have a lot of people with HTN in the society’ (P5).  *HTN is when a person has excess blood’ (P10)  * ‘When the blood in adults are more, it is called HTN’ ( P11);  *HTN means ‘more blood’ in my body, (P 17).  * I know it happens when the body has blood in excess. So, the type of food that gives us more blood should be reduced as we age so that we will not get hypertension” (P18). | More blood in the body |  |
| *‘Most people who have HTN are late eaters and they don’t eat maybe some small fruits to sleep (P4);  *For me I believe that eating heavy foods at night to sleep causes HTN because that makes the body still work like day time when you’re asleep (P6).  *This disease is caused by eating heavy foods at night time especially in our parents (P9);  *Eating at night time contributes to HTN, most people are too hungry in the evening time and they don’t also like to drink maybe tea because, they are too hungry (P25);  * a lot of people like to eat their heavy foods to get full and go to bed right after that, so even when they are asleep their bodies are still working like day time’  P20).  * We eat heavy foods during supper time. So, the heart overworks at night and may cause HTN (P12);  * Our dinner on campus is always late and heavy and we can get HTN since that makes the body do an extra job even when we sleep. (P14;).  * “for me, I know hypertension is pressure, so if there is too much pressure like work and exams periods you can become stressed and get hypertension” (P22). | * Time and type of food. | Perceived causes of HTN |
| *“There are too many synthetic oils in our meals nowadays and I think they can cause HTN (P3); *The oils in our meals are more synthetic and that can lead to HTN (P15).  * per its definition, I think we should reduce foods that produces more blood (P18);  * According to my understanding of HTN I think that blood producing foods should be reduced in our foods. (P5).  *I believe HTN can be caused by taking blood tonics (P17),  *For sure, blood tonics are a cause to HTN (P11). | * Dietary factors |  |
| *“When we have problems and we don’t tell others we can get hypertension. Especially, our mothers. Most of them think too much” (P16).  * Overthinking because of problems causes the pressure to rise (P22). | * Worry and unshared problems |  |
| * As for HTN, it’s a spiritual disease. Like diabetes, if you get it, you have to fast and pray for God to intervene, otherwise, it will be with you forever and if you get this HTN, you are likely to get diabetes too in the future” (P8).  * This disease I believe can be bought spiritually and given to someone for it to manifest physically, so I think when they tell you at the hospital that you have it, the first place to go is the church house” (P7).  * This is a spiritual disease like cancer (P10). | * Spiritual connotation |  |
| * when we stop eating sugar HTN will reduce because of the relationship it has with diabetes” (P24).  * For me, I believe artificial spices are the causes of hypertension in our time now. Most foods are artificially spiced, even at the school’s kitchen, but we can’t complain? If we stop or reduce artificial spices, it can reduce HTN and even diabetes” (P23).  * I personally believe that, when we eat a bit early in the evening the food will digest well and our bodies will also rest at night (P6).  *I agree with what my friend is saying “that artificial spices should be reduced and see if HTN will not be reduced in adults (P2).  * When we reduce the intake of fats and artificial spices which is mostly salty, HTN may be avoided. | *Avoidance of sugar and artificial spices. | Perceived knowledge on prevention of HTN |
| *I don’t actually know the meaning of the disease, but per its local name it suggests too much blood is a problem, so I think blood tonic should be avoided because it can give you more blood and then HTN will follow (P20).  *As for me, I suggest we eat foods to get blood rather than blood tonics, because some of them even when you take, your heart beats faster (P19). | *Avoidance of blood tonics |  |
|  | **Others** |  |
| I think exercising regularly is a good way to prevent HTN (P1) |  |  |
| I also think that a reduction in fats and oils foods plus artificial spices in our age group from now will prevent us from HTN in future (P3). |  |  |
